# Proteomics of Uveal Melanoma: A Minireview

**DOI:** 10.1155/2013/820953

**Published:** 2013-09-03

**Authors:** Søren K. O. Abildgaard, Henrik Vorum

**Affiliations:** Department of Ophthalmology, Aalborg University Hospital, Hobrovej 18-22, 9000 Aalborg, Denmark

## Abstract

Uveal melanoma (UM) continues to be associated with a high mortality rate of up to 50% due to metastatic spread primarily to the liver. Currently there are relatively effective treatments for the primary tumor, though the management of the metastatic disease remains inadequate. Conventional diagnostic tools have a low sensitivity for detecting metastasis, and early detection of metastatic spread would allow more treatment options that could ultimately increase survival of UM patients. Advanced proteomic methods have already helped to find potential biomarkers associated with UM pathogenesis and metastasis. In the present review we discuss the field of proteomics in relation to studies elucidating biomarkers of UM, where proteins such as S-100**β**, osteopontin (OPN), and melanoma inhibitory activity (MIA) have been shown to be associated with metastasis.

## 1. Introduction

Uveal melanoma (UM) is an ocular cancer involving the uveal tract (iris, ciliary body, and the choroid). The incidence is approximately 4.3 per million/year in the United States occurring mostly amongst white males [1]. With treatment UM has a 5-year-survival rate of 77–84% [2–4]. Prognosis depends on a number of factors including primary tumor size, time of diagnosis, and whether metastasis is present [5, 6]. Larger tumors have a mortality rate of approximately 50% shortly after diagnosis, and medium sized tumors also have a similar mortality rate when measured 15 years after primary tumor treatment [4, 7, 8]. Metastatic disease usually involves the liver and almost always leads to death within 15 months [6].

It is believed that that most choroidal, perhaps most uveal, melanomas origin from transformed benign naevus [9].

Clinically the primary diagnosis of UM of the choroid often involves decreased visual acuity and scotoma secondary to retinal detachment, with slit lamp biomicroscopy showing melanotic or amelanotic tumor with or without orange dusting. The diagnosis is often supported by an ultrasound investigation showing acoustic hollowness [9–11].

Primary tumor is treated with either brachytherapy using radioactive plaques in an effort to preserve the tissues of the eye or enucleation. There is no difference in survival rate, between these two treatments, for medium and large sized tumors [7].

Metastasis is searched for but seldom found at the primary diagnosis, which often develops in the following months to years [12]. So far the best methods for detecting metastasis are nonspecific liver enzymes serum concentration (lactate dehydrogenase (LD), alkaline phosphatase (AP), aspartate aminotransferase (ASAT), alanine aminotransferase (ALAT) and *γ*-glutamyl transpeptidase (*γ*-GT)), ultrasound, computed tomography (CT), and magnetic resonance imaging (MRI) [12, 13]. These methods have a sensitivity of 27–67% and a specificity of 90–96% with CT being significantly more sensitive than ultrasound and lactate dehydrogenase (LD) being the most sensitive serologic marker [13–15]. Treatments with liver resection, if there is only a small number of metastasis, have proven useful in increasing survival time 3.7-fold. Thus far, to our knowledge, chemotherapy has not been proven as an effective treatment in UM metastasis [12, 16].

There is an urgent need for early diagnosis of the primary tumor, as well as its metastatic spread, and for the development of improved methods of treatment [7]. Indeed it is likely that micrometastasis may be present in the majority of patients at the time of the primary diagnosis, but due to their small size and reduced cell turnover they are undetectable by conventional methods [18]. Therefore, there is understandably a great deal of research aimed at identifying primary and metastatic UM tumor biomarkers, to which discovery based proteomics has contributed significantly which we will discuss in this present review.

## 2. Proteomics

**Figure 1:**
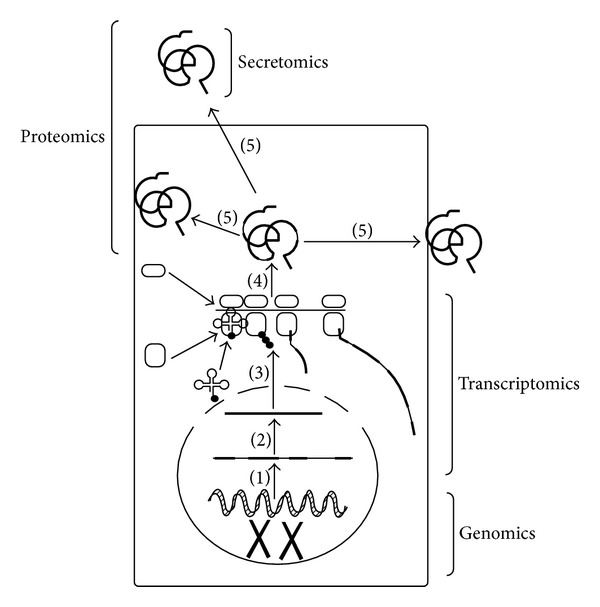
The various stages of protein production: (1) Transcription, (2) posttranscriptional processing, (3) Translation, (4) posttranslational modification, and (5) posttranslational processing and afterwards intra- and extracellular use of the proteins.

The search for tumor biomarkers and therapeutic targets has largely been centered on genomics and transcriptomic studies, though in the last few years proteomics has emerged as a strong player in this field. Proteomics was originally defined as the study of the total protein complement of the genome [19], but the definition has now broadened to include the study of single proteins and their variants and modifications [20]. Secretomics is a subgroup of proteomics, which studies the protein secretion from the cells that is measurable in the serum and represents an especially promising method to study the development of metastasis in patients with UM [21] (Figure 1).

### 2.1. Proteome Changes with Cancer Evolution

Normal cells protect their genome from damage by a complicated caretaker system, which involves enzymes that monitor and repair DNA and act at certain checkpoints during mitosis. Cells can become cancerous following changes in the normal cellular genome, which enables the cell to avoid the caretaker system by a number of ways such as changes in growth factor response, apoptosis regulation, and ability to spread throughout the tissues and body (Table 1). A malignant tumor comprises of a mixture of cancerous and normal cells. This enables the cancerous cells to be oxygenated, nourished and near capillaries to ease migration. The growth of the normal cells is probably stimulated by “growth signals” from the cancerous cells [22]. The change in gene expression is converted to a change in the tumor cells proteomic profile enabling the tumor to grow and spread [23]. In the following segments, we systematically review the current proteomics studies, seen in the light of the cancer process described above many of the proteins depicted below falls into these categories.

### 2.2. Proteomics of Nevi Transformation

It is believed that most cases of UM develop from a uveal nevi transformation [9]. The changes in protein profile when a nevus changes into a choroidal melanoma were investigated by Bande et al. [24] who examined serum concentrations of an oncoprotein DJ-1 in 53 nevi patients and 32 healthy controls with the enzyme-linked immunosorbent assay (ELISA) method. Parkinson protein no. 7 (PARK 7) also known as DJ-1 is an oncogene which is transcribed into an oxidative stress response protein (DJ-1); this protein defends the cell against oxidative species. Bande et al. showed that DJ-1 concentrations were not significantly elevated in nervi patients when compared to the controls (37,39 ng/mL versus 32,98 ng/mL). But when they examined concentrations of nervi patients with ocular symptoms at time of UM diagnosis (69,79 ng/mL), ultrasound diagnosed acoustic hollowness (86,95 ng/mL), nevus thickness > 1,5 mm (73,22 ng/mL), and large basal diameter > 8 mm (73,03 ng/mL) and compared them to nervi patients without symptoms and objective findings, they found a statistically significant elevated DJ-1 concentration. Three of the 53 patients had a nevus transformed into a UM during the studied period. The baseline average serum level of these patients was 89,56 ng/mL compared to the nevus group (37,39 ng/mL) and the control group (32,98 ng/mL). Though the number of the studied groups was small with very few nevus transformations, DJ-1 could be a useful biomarker in predicting nevus transformations and indeed has also been shown to be elevated in breast cancer [25].

### 2.3. Proteomics of Uveal Melanoma Cell Culture

**Figure 2:**
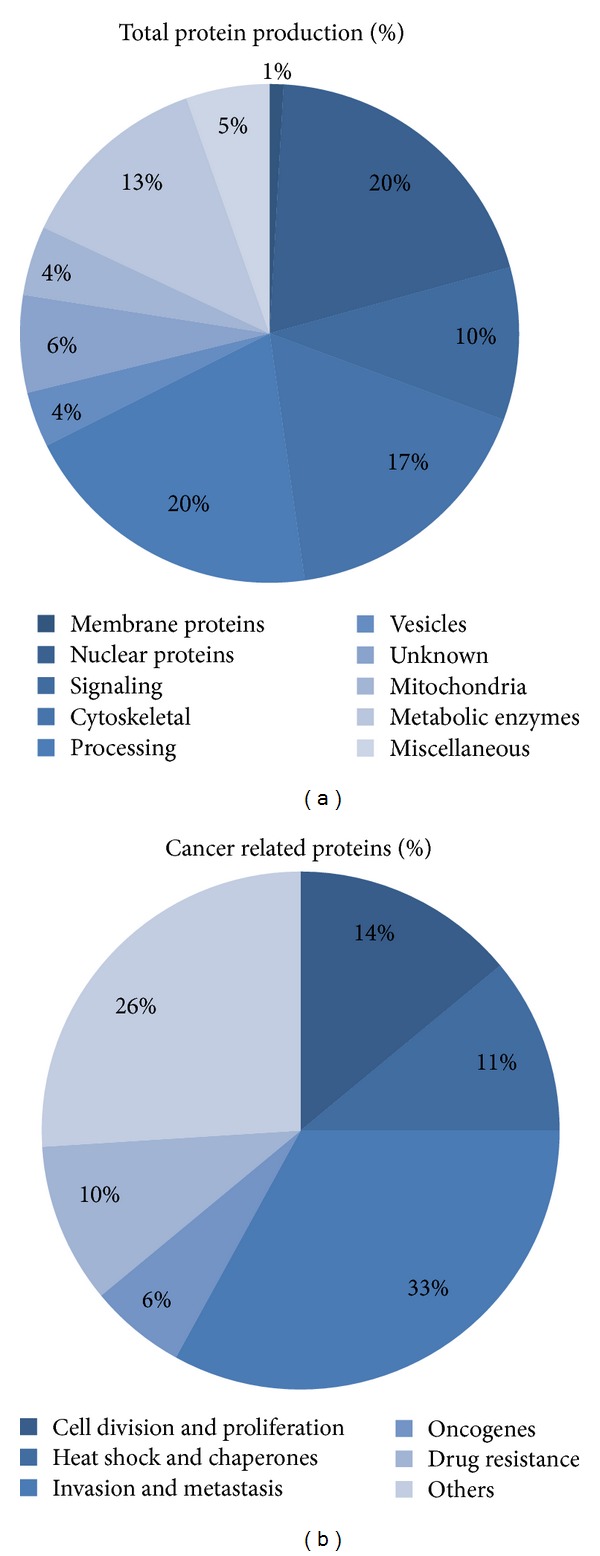
Total and cancer related protein production of UM cell culture divided by their function. Adopted from Pardo et al. [17].

The protein profile of a single UM cell culture was investigated by Pardo et al. in 2005 [17]. The investigators used two-dimensional gel electrophoresis (2-DE) and mass spectrometry (MS) to analyse the protein in human UM cell culture. The study identified 683 proteins from 393 different genes. About 69 of these proteins were related to the carcinogenesis as shown in Figure 2. This is of course a momentary picture of a protein profile which is in constant change; however, their findings appear to greatly correlate with previous studies [23]. The proteins found include Ezrin which is involved in connecting the cytoskeletal to the plasma membrane and has a role in cell division and proliferation. A number of heat shock proteins (HSP), including HSP 90, help with protein folding thereby functioning as a chaperone and defending the protein from damage such as that originating from oxidative stress. Pardo et al. [17] also found proteases and peptidases involved in degeneration of extra- and intracellular proteins and matrix, which include the family of cathepsins, thereby helping the invasion and metastasis process; this also includes the S100 proteins (discussed later). Furthermore, oncoproteins such as DJ-1, which defends the cell against oxidative species, and proteins that conferred resistance to cancer drug treatment were also found.

What is quite striking is that 33% of the 69 cancer related proteins were involved in tumor invasion and metastasis. Though UM tumors are in constant change reflecting their genetic and subsequent protein expression, this momentary picture gives an indication of how much efforts are necessary for the cancer cell to change its surroundings and the intracellular environment [17]. 

### 2.4. Proteomics in Diagnostic Markers of Uveal Melanoma

As mentioned previously the diagnosis of uveal melanoma is mostly based on clinical observations, and there is often a need to distinguish atypical melanoma such as amelanotic/hypomelanotic melanomas and other types of ocular tumors including ocular metastasis and retinal detachments with subretinal hemorrhages or exudation. Therefore, one of the main aims of proteomics is to establish a biomarker that can be used along with the clinical and histological presentation of the tumor [26]. 

One of the earliest studied biomarkers is the acidic protein S-100, which is a dimer comprised of two almost identical subunits named *α* and *β*. This marker has been shown immunohistochemically to be present in 70% to 91% of primary tumor biopsies and UM cell cultures with the strongest correlation with S-100*β* subunit [26–29]. S-100 protein has been highly elevated in all ocular melanomas, inversely proportionally to the amount of melanin. It is not consistently accumulated in ocular fluids [24] or in serum, where it to our knowledge has not proven useful as a diagnostic or prognostic marker [29]. S-100 is not a unique marker for UM and is also changed in many other tissues and tumors including cutaneous melanomas. This makes it unsuitable for fluid screenings and may also question its usefulness as a histopathologic marker [26]. 

Other protein markers investigated include HMB-45 which has been found in approximately 93 to 100% of all UM, correlated to melanocytic tumors [27, 30] and melanoma inhibitory activity (MIA) found in 4 out of 5 primary tumors [31]. 

p53 is a well known modulator of cellular proliferation, and it has also been shown to inhibit angiogenesis in the normal cell [32]. The relationship between p53 expression in UM was investigated by Chowers et al. in 98 UM tissue blocks [33]. It was found that increased p53 expression was significantly associated to tumors with high proliferative activity and epithelioid cell type, though not related to other microcirculating patterns (normal blood vessels, tumor silenced area, loops, cross-linking, and much more) previously shown to be associated with death from UM metastasis. These results have also been shown by other studies [33–35]. It is now believed that p53 mutation is associated with a high proliferative activity [33]. 

As an involvement of previous work Pardo et al.[32] studied the proteomics of two in vitro cell lines with different tumor progressive status before (UM < 7) and after 7 passages (UM > 7) using 2-DE and MS. They found that UM < 7 contained more actin related and actin-binding proteins, such as vimentin and adhesive proteins such as Ezrin, when compared to UM > 7. In contrast, UM > 7 had the presence of proteins related to disassembly of actin filaments, in addition to an increase in glycolytic enzymes, such as alpha enolase. Some proteins decreased or disappeared, while new proteins, namely, high mobility group protein 1 (HGM-1), were identified in the UM > 7 cell line. They also found proteins expressed in UM > 7 that promoted cell survival such as HS1-binding protein and new adhesive proteins such as melanoma associated antigen 18 (MUC18). They identified HMG-1, which is involved in transcription of genes in the metastatic cascade, in different human cancers, and MUC18, a cell adhesive molecule (CAM) involved in endothelium adhesion, that may be involved in allowing hematogenous spread. Both HMG-1 and MUC18 are suggested as potential invasive markers as they are expressed only in UM > 7 and not in UM < 7. Finally, DJ-1 oncogene was identified as a potential biomarker for UM as it is present in all UM cell lines (including UM < 7 and UM > 7) though not in non-UM cell lines. DJ-1 was confirmed in the UM cell culture medium by western blot (WB), thereby identifying it as a secretory molecule. 

Pardo et al. [36] also used WB and MS to identify potential biomarkers in UM. By studying UM cell line secretomes and autoantibodies against secreted UM protein in 11 patients, they identified three potential biomarkers. Cathepsin is a lysosomal acid proteinase that was overexpressed in the UM cell line compared to non-UM cell line, even more in UM < 7 passage compared to UM > 7 passages. It was also detectable in UM patients sera. The same was found with melanoma specific antigen gp100 and adapter protein syntenin 1, though syntenin 1 was not detected in patient sera.

### 2.5. Proteomics in Metastasis of Uveal Melanoma

The proteomic profiles of metastasis compared to primary tumor in UM were investigated by Zuidervaart et al. [37]. They compared the proteomic profiles of 2 liver metastasis to those of the primary UM cells with the use of 2-DE and MS. Out of 1184 spots on the gels they identified 24 proteins that differed in metastasis when compared to primary tumor. They found no difference between the two metastatic cell lines but an increase in cellular defense, apoptosis, proliferation, and migration. They also found a downregulation of metabolic proteins when compared with the primary tumor. As depicted in Table 1, the proteins shown to increase include heat shock protein 27 (HSP-27), involved in protein conformation stabilization, and Cathepsin Z, involved in lysosomal proteolysis, while the production of proteases is generally decreased compared to primary tumor. This study gives some important insight into the differences between primary tumor and the metastasis (Table 2).

The proteomic differences between metastasizing UM and nonmetastasizing UM have also been investigated by Linge et al. [38]. Instead of comparing the primary tumor to the metastatic, they looked at 25 primary UM patients with a minimum followup of 7 years and subsequently identified 9 patients who developed metastatic disease. They used 2-DE and MS to identify 14 proteins which significantly differed in amount between the two groups (Table 3). Amongst those identified they made an in vitro function study by silencing the fatty acid-binding protein, heart-type (FABP3), and triosephosphate isomerase (TPI1) gene. Their results showed that significantly less cells invaded through the underlying membrane but also a significant decrease in cell motility was found.

As mentioned earlier the leading cause of death in UM patients is metastasis that is primarily to the liver. Although micrometastasis at the time of the primary diagnosis is thought to be present, most metastasis is usually only found later at more advanced stages of UM disease. In recent years proteomic investigations have been used to try and identify biomarkers that could help to determine which UM patients have micrometastasis at the time of primary diagnosis and have increased risk of developing metastasis, and therefore those patients that are in need of adjuvant treatments including liver resection. 

The proteomic search can be divided into two main approaches. Firstly, those studies upon the primary tumor to try to determine if there is metastasis at the time of enucleation (all these studies performed on enucleated tumors); and secondly, those studies on the proteomic expression of the serum, vitreous or aqueous humour associated with the primary tumor and/or its metastasis. Later we discuss the potential biomarkers that have been investigated using these two methods.

### 2.6. Proteomics as Histopathologic Markers in UM Metastasis

As shown in Table 4 a number of potential biomarkers associated with UM metastasis have been identified, most of them investigated by immunohistochemistry (ICH). Many of them have shown a significant change related to metastasis. Some of the most promising candidates include melanoma cell adhesion molecule (MCAM), stem cell factor (SCF), HSP-27, and vimentin [39–41] since these proteins have a crucial role in normal, as well as pathological cell functions and can be found within the primary tumor. Coupland et al. [40] use prior knowledge from the genomics field and search for monosomy 3 which is significantly associated with hepatic metastasis in UM [42, 43].

### 2.7. Proteomics as Serum Biomarkers in UM Metastasis

During the last couple of years there has been an extensive search for serum biomarkers, since early diagnosis is the key to successful treatment of metastatic UM. As mentioned earlier, current blood biomarkers and imaging techniques have a sensitivity of 27–67%, with computed tomography (CT) and lactate dehydrogenase (LD) being the most sensitive [13, 15, 44]. Often the metastatic burden is too great at the time of the diagnosis, and early diagnosis allows more treatment options that give better chance of curing the patient [16, 18]. Studies show that melanoma inhibitory activity (MIA), osteopontin (OPN) and tissue polypeptide-specific antigen cytokeratin 18 (TSP) are significantly elevated in serum of metastatic UM patients when compared with nonmetastatic patients (Table 5). They more than double in measurable concentration in serum, and a threshold has even been suggested for MIA of 8,3 ng/mL giving it a sensitivity of 82% and a negative predictive value of 97% as shown by Klingenstein et al. [45], which is better than conventional serum markers. The size of the tested groups (503 UM patients in Klingenstein et al. [45]) also makes it ready for clinical trials, and it is likely that the same goes for OPN and maybe even for TSP. Vascular endothelial growth factor (VEGF) has not been shown to have a large increase with metastasis as one might expect, due to the wide interpersonal variability in serum VEGF levels [46]. Barak et al. [46] suggest that this may be due to the fact that VEGF has two very different splicing products that occur naturally. These two VEGF products have very different abilities regarding pro- and antiangiogenic signals, and a change in ratios VEGF types cannot be distinguished by current tests such as ELISA.

### 2.8. Proteomics as a Prognostic Marker in UM

UM has a poor prognosis, especially in the metastatic stage. Prognostic factors include clinical and histomorphological appearance (tumour size, the relation to surrounding tissue, cellular type, etc.), in addition to monosomy of chromosome 3 that is associated with a decreased survival due to hepatic metastasis [42, 43]. In recent years Jmor et al. [47] have investigated the prognostic value of HSP-27, a well known chaperone involved in cell proteolysis inhibition. Jmor et al. [47] perform IHC upon enucleated eyes of 99 UM patients, 44 of which were predicted dead within a follow-up period of 8 years by conventional methods. They only identified 5 deaths due to metastatic disease and 5 patients with developed metastasis all of which were monosomy 3 positive of which 6 were found to have a low to negative HSP-27 score (score ≤ 6). Jmor et al. [47] calculated the prognostic value of HSP score by using a mathematical analytic model. They found that HSP-27 ≤ 6 score significantly predicted a decreased < 8 years survival rate and when combined with clinicopathological factors predicted monosomy 3 with a sensitivity of 78% and a specificity of 72% [40, 42, 43, 47].

## 3. Future Perspectives

 Healthy cells become cancerous through genetic mutations, which are implemented through splicing in effector proteins found intra- as well as extracellularly including vitreous body and serum. Through proteomic analysis these proteins may be identified in primary tumor, ocular fluids, and in the patient's serum, thereby assisting in diagnosis, primary tumor classification, and metastatic identification, together with conventional methods. The proteomic profiles of two UM cell lines and tissue samples are never identical, but as proteomic studies have shown us there are many similarities, which may make it possible to one day identify clinically useful protein biomarkers and therapeutic targets. 

Many of the current biomarkers have only been found in small pilot studies and are in need of validation in larger prospective patient cohort studies. This has proven to be difficult given that the incidence of UM is quite low compared to that of other tumors and that the followup of metastatic patients has proven difficult because of large mortality rates as described by Reiniger et al. [48]. However, this task is essential if potential proteomic markers will have a chance to be implemented in everyday clinical assessments. 

There is an especially important need for further diagnostic aids in nevi-transformation, atypical melanoma, micro- and macrometastasis. Serum biomarkers have an obvious advantage over histopathological biomarkers in that it is not necessary to obtain tissue samples from the primary tumor or metastasis. When it comes to diagnosing metastasis at the time of primary UM treatment, serum biomarkers can be used as a prospective tool to monitor the UM patients, while histopathologic biomarkers of primary UM biopsies can be used for prognostic staining and to identify those UM patients of high risk or high probability of micro- and macrometastasis and therefore in need of additional treatments. 

Studies performed with the potential UM biomarkers of MIA and OPN show especially promising results in terms of increased sensitivity of detecting UM metastasis compared with conventional methods [45, 49]. However, there remains a need for an in-depth understanding of the UM metastasizing process and the identification of further potential biomarkers with proteomics centered upon

1. characterization of primary UM at differing stages including metastatic UM
2. prospective studies of biomarkers in UM patients using quick and hopefully cost effective tests
3. implementation into effective clinical practices.

Optimal UM management may be to combine proteomics, genomics, and transcriptomics in order to identify high risk UM patients and establish their surveillance together with early and individualized treatment. Furthermore, as proteomic analysis becomes more cost effective and rapid in the future, it may even be possible to ascertain an individual set of biomarkers for each UM patient. However, this will require better knowledge of UM development to which proteomics studies will also surely continue to contribute.

## Figures and Tables

**Table 1:** Cellular changes towards cancerous development and spread as described by Hanahan and Weinberg [22].

(1) Self-sufficiency in growth signals—changing the intra- or extracellular response to existing growth factors, increasing the production of growth factor via self-production or by stimulating neighboring cells.	
(2) Insensitivity to antigrowth signals—changing of growth factor receptors or signaling pathways.	
(3) Antiapoptosis—changing the intra- or extracellular sensors that induce apoptosis such as tumor suppressor protein 53 (p53).	
(4) Sustained angiogenesis—production of vascular endothelial growth factor (VEGF) and other factors.	
(5) Unrestrained replicative potential—production of telomerases ensuring that the telomeres on the chromosomes avoid normal progressive shortening during abnormally increased cell cycles.	
(6) Tissue invasion and metastasis—reducing adhesion to other cells and the extracellular matrix by decreasing CAMs (adhesion molecules), calcium dependent cadherin families, especially E-cadherin and integrins. Increasing protease productions from the tumor, stromal or immunological cells enables tumor spread. The cells have to adhere to the new tissue by expressing adhesive molecules.	

**Table 2:** Protein change in the metastatic UM cell line.

Category/name	Function	Change (*x*-fold)
Cellular defense		
HSP27	Protein stabilisation	++
Apoptosis/degeneration		
Cathepsin Z	Lysosomal proteolysis	++
Proliferation		
Annexin	Growth factor	++
Migration		
Cofilin	Actin turnover	+++
Tropomodulin 3	Actin turnover	++++++
CLIM1	Actin kinase adaptor	++++++
Galectin	Cell to cell interaction	++
*β*-Hexosaminidase *β*-subunit	Glycosidasis	++++++
Metabolism		
Pyruvate kinase 3	Glycolysis	−
Enolase 1	Glycolysis	−
Nuclear transport		
Ran-binding protein 1	RanGAP activity	++
elF5A	Cofactor in nuclear export	++++++
Translation		
CRHSP-24	Translation regulating protein	++++++

Adapted from Zuidervaart et al. [37] (+) 0–1.5. (++) 1.5–5. (+++) 5–10. (++++) 10–50. (+++++) 50–100. (++++++) >100-fold increase. Minus depicts a decrease.

**Table 3:** Differences in protein levels between primary UM with and without subsequent metastasis.

No.	Gene name	Protein name	Average ratio	*t*-test
1	PDIA3	Protein disulfide-isomerase A3 precursor	1,5	0,011
2	VIM	Vimentin	1,8	0,007
3	SELENBP1	Selenium-binding protein 1	1,3	0,044
4	ENO1	Alpha-enolase	1,4	0,007
5	EIF2S1	Eukaryotic translation initiation factor 2 subunit 1	−1,6	0,035
6	CAPZA1	F-actin capping protein subunit alpha-1	1,3	0,028
7	PSMA3	Proteasome subunit alpha type 3	−1,2	0,026
8	RPSA	40S ribosomal protein SA	−1,4	0,023
9	ERP29	Endoplasmic reticulum protein ERp29	1,4	0,040
10	TPI1	Triosephosphate isomerase	1,7	0,00009
11	PARK7	Protein DJ-1	1,2	0,018
12	TUBB	Tubulin beta chain	−1,7	0,017
13	TUBA1B	Tubulin alpha-1B chain	−1,9	0,006
14	FABP3	Fatty acid-binding protein, heart	2,2	0,00035

A selection of proteins differentially expressed between primary UM with and without metastasis. Adopted from Linge et al. [38]. Ratios were calculated as the difference between primary UM that subsequently metastasized versus primary UM that did not metastasize.

**Table 4:** Histopathologic proteomic markers in metastatic UM (explanations below).

Name	Authors	Results	Function	Number	Method	Year
S-100	Luyten et al. [31]	Present in 66% of UM metastasis	Cytoskeletal	29 UM and metastasis	IC	1996

HMB-45	Luyten et al. [31]	(1) Present in 91% of UM metastasis. (2) Significantly more sensitive than S-100 (*P* = 0,04)	Unknown	29 UM and metastasis	IC	1996

NKI-C3	Luyten et al. [31]	Present in 71% of UM metastasis	Unknown	29 UM and metastasis	IC	1996

MIA	Schaller et al. [50]	4 out of 5 primary UM (80%) and 6 out of 8 metastasis (80%) were found positive for MIA; in the last two metastasis detection was not possible	Cell-matrix adhesion inhibitor	5 FFPE UM, 8 FFPE metastasis	IC	2002

NSB1	Ehlers and Harbour [51]	High expression was significantly correlated to a low survival rate (22% versus 100% *P* = 0,01)	DNA damage repair	49 UM, 4 deaths	Gen. + IC	2005

c-Met	Mallikarjuna et al. [52]	High expression related to liver metastasis (*P* = 0,0009), speculation is that it is ligand independent as HGF was not elevated	Hepatocyte growth factor (HGF) receptor involved in HGF's stimulation of tumor cells	60 UM, 6 metastasis	IC	2007

VEGF-A and MMP-9	Sahin et al. [53]	Not present in normal ocular tissues. Significantly elevated in lymphocytic infiltration, necrosis, EMP (loop and/or network) formation. VEGF was significantly elevated in metastasis (*P* = 0,02); MMP-9 was almost significantly elevated (*P* = 0,052). Metastasis not correlated to cell type, tumor seize, mitotic rate, degree of pigmentation, necrosis, and EMPs	VEGF-A = most important proangiogenic factor MMP-9 = metalloproteinase, Zn binding, key role in angiogenesis	100 UM, metastasis 14, deaths 2	IC	2007

E-cadherin + VE-cadherin + Hif1*α*	Chang et al. [54]	Significantly associated with class 2 tumor type (a significant predictor of metastasis *P* = 0,0001) and epithelioid cell type (*P* < 0,001)	Not described in the paper	29 class 1 tumors and 28 class 2 tumors	IC	2008

MCAM (=MUC18/Mel-Cam/CD146)Φ	Beutel et al. [39]	Increased in primary tumor with metastasis compared to nonmetastatic tumor (from 32,5% to 81,8%, *P* = 0,0001)	Adhesion molecule expressed on vascular endothelium, believed to be involved in tumor migration through the endothelium and vascularization	35 UM, 16 metastasis	IC	2009

HSP-27	Coupland et al. [40]	Decreased in densitometry with monosomy 3 (10,51–6,67 ODxArea *P* = 0,005)	Chaperone involved in heat and stress protection	41 FFPE UM, 20 monosomy 3	MS IC WB	2010

Vimentin	Coupland et al. [40]	Increased in densitometry with monosomy 3 (5,82–12,20 ODxArea *P* = 0,003)	Cytoskeletal component	41 FFPE UM, 20 monosomy 3	MS IC WB	2010

SCFΦ	Lüke et al. [41]	Decreased in expression in primary tumors with systemic metastasis (from 77,2% to 30,1%, *P* < 0,0001)	Ligand to the c-Kit receptor tyrosine kinase has a crucial role during defined stages of the development of mature melanocytes	35 UM, 16 metastasis	IC	2011

c-KitΦ	Lüke et al. [41]	No significant correlation with systemic metastasis	Receptor tyrosine kinase has a crucial role during defined stages of the development of mature melanocytes	35 UM, 16 metastasis	IC	2011

LZTS1 + *β*-catenin	Simões et al. [55]	When combined *β*-catenin negative plus any LZTS1 expression was significantly related to metastatic death	LZTS1 = tumor suppressor *β*-catenin = possibly a role in promoting tumorigenesis	82 FFPE UM, 27 dead of metastasis	IC	2011

MS: mass spectrometry, IHC: Immunohistochemistry, WB: western blotting, Mono.: monosomy, Φ: prognostic marker, MIA: melanoma inhibitory activity, NSB1: Nijmegen breakage syndrome 1, MCAM: melanoma cell adhesion molecule, HSP-27: heat shock protein 27, SCF: stem cell factor, LZTS1: leucine-zipper tumor suppressor 1, VEGF-A: vascular endothelial growth factor, MMP-9: matrix metalloproteinase 9, VE: vascular endothelial, Hif1*α*: hypoxia-inducible factor 1 *α*.

**Table 5:** Proteomic biomarkers in metastatic UM (explanations below).

Marker	Who/year/method	Results	Function	Number	Materiel
S-100-*β*	Missotten et al. [56]/2003/ Immuneluminometric assay	No significant correlation with any of the investigated factors, including metastasis *P* = 0,96	Unknown	64 UM patients, 20 metastasis	Serum

S-100-*β*	Missotten et al. [57]/2007/ Immunoluminometric assay	Not related to any prognostic parameter, significantly elevated in metastatic UM (median 0,7 to 0,23 *μ*g/L, *P* < 0,001), along with LD better than MIA and *γ*-GT	Calcium-binding protein in the calmodulin/troponin C superfamily	194 UM patients, 30 metastasis	Serum

MIA	Schaller et al. [58]/2002/MIA ELISA	Increased in metastatic UM (median of 6,6 to 26,28 ng/mL, *P* < 0,001) and metastasis development (median of 6,6 to 29,2 ng/mL, *P* < 0,001) Not a prognostic parameter for size and survival (NB metastasis results more scattered than nonmetastatic)	Induces cell-matrix detachment because it binds to fibronectin and laminin	139 UM patients, 8 metastasis (3 developed), 61 followed over time	Serum

MIA	Reiniger et al. [48]/2005/MIA ELISA	Increased in metastatic UM (median of 6 versus 13,03 ng/mL, *P* < 0,001) and in their development (5,92 to 12,21 ng/mL, *P* = 0,005) Not a predictive marker for tumor height and treatment (NB metastasis results more scattered than nonmetastatic)	Induces cell-matrix detachment because it binds to fibronectin and laminin	305 UM patients, 20 metastasis (8 developed)	Serum

MIA	Missotten et al. [57]/2007/MIA ELISA	Not related to any prognostic parameter, significantly elevated in metastatic UM (*P* < 0,01), not as good as S-100-*β* and LD	Induces cell-matrix detachment because it binds to fibronectin and laminin	194 UM patients, 30 metastasis	Serum

MIA	Haritoglou et al. [49]/2009/MIA ELISA	Increased in metastatic UM (median 5,64 to 13,11 ng/mL, *P* < 0,001) and developed metastasis (9,8 to 26,53 ng/mL, *P* < 0,001)	Induces cell-matrix detachment because it binds to fibronectin and laminin	32 UM patients, 14 metastasis (1 developed)	Serum

MIA	Klingenstein et al. [45]/2011/MIA ELISA	Increased in metastatic UM (median 6,97 to 11,69, *P* < 0,001), ROC analysis with AUC = 0.84 giving a suggested *MIA threshold of 8,3 ng/mL* (NB: related to ELISA kit) with a sensitivity of 82% and a specificity of 77%, positive predictive value 0,30 and a negative predictive value of 0,97. Metastasis development showed an increase (median of 6,8 to 19,6 ng/mL)	Induces cell-matrix detachment because it binds to fibronectin and laminin	503 UM patients, 54 metastasis, 28 developed metastasis during followup	Serum

OPN	Kadkol et al. [59]/2006/OPN mRNA, IC and serum OPN ELISA	OPN mRNA was increased in highly invasive primary and metastatic UM (6- and 250-fold) Histologically it was related to vasculogenic mimicry, not macrophage presence. Serologically it was significantly increased in metastatic UM versus 10-Y DF (median 17,62 versus 7,15 ng/mL, *P* = 0,0001) and pre- versus postmetastasis (6,19 versus 19,66 ng/mL, *P* = 0,0004) with a cutoff of 10 ng/mL sensitivity and specificity of 83,7% and ROC with AUC being 96% (the probability of correct diagnosis of metastasis)	Component of the noncollagenous bone matrix, actively promotes tumoregenic phenotype and contributes to metastasis	3 UM cell lines, serum from 37 10-Y DF, 15 metastatic (8 with pre- and postmetastatic sampling)	Tissue + Transcriptom + Serum

OPN + S-100*β* + MIA	Barak et al. [60]/2007/ELISA + ROC curve with AUC	Increased metastatic versus 10-Y DF in OPN (8-9 to 14–18 ng/mL, *P* = 0,0037), S-100*β* (4–6 to 12–14 *P* = 0,0111), and MIA (0,05–0,1 to 0,325–0,45 ug/L, *P* = 0,0005). Development of metastasis OPN (4-5 to 18–23 ng/mL, *P* = 0,002), S-100*β* (6-7 to 17–22 ng/mL, *P* = 0,046), and MIA (0,03–0,06 to 0,12–0,18 ug/L, *P* = 0,045) AUC = 91%	Se under the specific markers	38 patients 10-Y DF (8 before/after metastasis)	Serum

OPN	Haritoglou et al. [49]/2009/ELISA	Increased in metastatic UM (median 47,39 to 152,01 ng/mL, *P* < 0,001) and developed metastasis (118,67 to 375,54 ng/mL, *P* < 0,001)	Actively promotes the tumorigenic phenotype and contributes to metastasis	32 UM patients, 14 metastasis amongst those 1 developed	Serum

VEGF	Barak et al. [46]/2011/VEGF ELISA	To wide interpersonal variability to show any significant difference between the groups. Ratios showed a significant increase from after treatment to 3 years after diagnose of 53%, but this disappeared after excluding two outliners	Vascular endothelial growth factor stimulating vascularization	23 UM, 58 10-year DF, 39 metastatic (17 patients before/after)	Serum

TSP	Barak et al. [61]/2007/Direct injection of UM cells (MUM2B) into mice (ELISA)	TSP was markedly elevated in MUM2B injected mice versus noninjected (84,7 U/L versus 601 *μ*g/L) In humans TSP was significantly elevated in metastatic versus 10-Y DF and controls (139,63 versus 69,29 and 54,23 U/L, *P* < 0,01).	TSP = tissue polypeptide-specific antigen cytokeratin 18	15 injected mice, 64 UM 10-Y DF, 37 metastatic (TSP before/after metastasis), 53 controls	Serum

MIA: melanoma inhibitory activity, OPN: osteopontin, VEGF: vascular endothelial growth factor, TSP: tissue polypeptide-specific (TSP) antigen cytokeratin 18, ROC: receiver operator characteristics, AUC: area under the curve, 10-Y DF: 10-year disease free.
